# Protective Effects of 17-βE_2_ on the Primary Hepatocytes of Rainbow Trout (*Oncorhynchus mykiss*) Under Acute Heat Stress

**DOI:** 10.3390/antiox13111316

**Published:** 2024-10-29

**Authors:** Guiyan Zhao, Zhe Liu, Junhao Lu, Jinqiang Quan, Yucai Pan

**Affiliations:** Department of College of Animal Science and Technology, Gansu Agricultural University, Lanzhou 730070, China; 18893812949@163.com (G.Z.); lujh@st.gsau.edu.cn (J.L.); quanjq@gsau.edu.cn (J.Q.); panyc@st.gsau.edu.cn (Y.P.)

**Keywords:** rainbow trout, heat stress, 17-βE_2_, estrogen receptors

## Abstract

The rainbow trout (*Oncorhynchus mykiss*) is a typical cold-water species. However, due to global warming, it has experienced prolonged high-temperature stress. Research indicates that thermotolerance in rainbow trout varies by sex at multiple physiological levels. Specifically, females exhibit higher thermotolerance, which may be attributed to estrogen-mediated signal transduction pathways. This study involved culturing primary hepatocytes from rainbow trout and exposing them to estradiol and estrogen receptor antagonists to assess estradiol’s protective effects. The analysis focused on expression of ER, HSPs genes, hepatocyte viability, and antioxidant indices. Four experimental groups were treated with 17-βE_2_ at concentrations of 0, 0.1, 1, and 10 μM/mL for durations of 4, 8, 12, 24, and 48 h at 18 °C. 17-βE_2_ treatment led to increased hepatocyte viability and enhanced SOD, GSH-Px, and CAT levels but decreased MDA levels. *hsp70a*, *hsp90β*, *era1*, and *erβ1* levels were notably higher, with the optimal 17-βE_2_ concentration being 1.0 μM/mL. Following heat stress (24 °C), the addition of 1.0 μM/mL 17-βE_2_ improved hepatocyte viability and increased SOD, GSH-Px, and CAT levels, while MDA content initially decreased before rising. The gene expression of *hsp70a*, *hsp90β*, *era1*, and *erβ1* was significantly elevated compared to controls. Flow cytometry analysis showed increased apoptosis after heat exposure; however, 17-βE_2_ treatment significantly reduced the heat stress-induced effects (*p* < 0.05). In conclusion, 17-βE_2_ and mild heat stress collaboratively enhanced the expression of HSPs and estrogen receptors, thereby providing protection to hepatocytes from heat stress damage, indicating a beneficial protective role of estradiol in rainbow trout hepatocytes.

## 1. Introduction

Global warming leads to seasonal increases in water temperatures and extreme heat wave events. Severe and irreversible impacts on the survival and function of poikilothermal animals have been reported [1]. These effects primarily manifest as metabolic disturbances [2], growth performance inhibition, tissue structural damage, and changes in gene expression associated with heat shock, immunity [3], and antioxidants [4], impacting the ability to scavenge reactive oxygen. Consequently, high-temperature stress has become a growing environmental challenge for the aquaculture industry, with particularly pressing implications for species that have long been adapted to low temperatures [5].

The rainbow trout (*Oncorhynchus mykiss*) is endorsed by the Food and Agriculture Organization (FAO) of the United Nations as an excellent cold-water fish due to its flavorful meat and high nutritional value. Its significant role in poverty alleviation and the development of local specialty industries in the western mountainous regions has led to its classification as a key advantageous industry for growth in Gansu Province. However, as aquatic ectotherms, rainbow trout thrive at an optimal water temperature of 12–18 °C and exhibit increased mortality when water temperatures exceed 25 °C [6,7]. Therefore, high summer temperatures are a serious constraint to rainbow trout culture. Previous studies have demonstrated that heat-stressed rainbow trout rely mainly on up-regulating heat shock protein (HSP) expression to increase their tolerance to heat stress. HSPs are molecular chaperones that can maintain protein stability and normal cellular function by assisting in the proper folding of proteins and preventing their misfolding. HSPs also enhance the repair of misfolded proteins, mitigate heat stress-induced protein damage, and protect cells from heat injury [8,9].

Our team focused on the heat stress mechanism in rainbow trout. We previously reported obvious sex differences in many of the heat stress indicators within rainbow trout. For example, in vivo thermal stimulation experiments revealed that the survival rate of females was significantly better than that of males. In particular, under conditions of extreme heat treatment (26 °C), all the fish that survived were females. In addition, at the serum biochemical level, serum indicators of oxidative stress also revealed that females had greater antioxidant capacity. More importantly, we also reported that the “estrogen signaling pathway” was an important pathway involved in the enrichment of differentially expressed genes and proteins prior to and following heat stress [10]. These findings revealed sex differences in heat tolerance in rainbow trout. However, how do sex differences in thermotolerance develop, and what are their specific mechanisms of action? To date, no systematic research has been conducted. This mechanism is not only of great scientific importance but also has great application value in terms of seed production, feed development, summer management, and other production processes.

Many studies have shown that steroid hormones, in which estrogen and estrogen receptors (ERs) play major roles, are major contributors to sex-related differences in phenotypic traits in animals [11,12]. Estrogens primarily include 17-β estradiol (17-βE_2_), estrone, and the metabolite estriol, with 17-βE_2_ being the most biologically active [13]. Based on studies of sex-related trait differences in other animals and previous research on heat stress in rainbow trout, it is hypothesized that estrogens, particularly 17-βE_2_ and its receptor-mediated signaling pathways, are important for the sex differences in heat tolerance of rainbow trout. The primary pathway involves estrogen competitively binding to ERs on HSPs, leading to the dissociation of HSPs and activation of their anti-heat stress functions [14]. Members of the HSP90 family are molecular chaperones for steroid hormone receptors and are involved in their stabilization and functional regulation [15,16], while the HSP70 family plays a crucial role in protein folding and translocation [17,18]. ERs interact with HSPs [19] to form chaperonin complexes that stabilize HSPs in an inactive state [20,21]. Therefore, when there is no estrogen, HSPs bind to ERs, forming the HSP-ER complex and suppressing the anti-thermal function of HSPs. Elevated estrogen levels in vivo promote the dissociation of HSPs from the HSP-ER complex by competitively binding ERs to HSPs, thus restoring the anti-heat stress activity of HSPs. Estrogen-mediated dissociation of HSPs not only enhances their thermal protective effects but also may activate estrogen-mediated signaling pathways, initiating various biological effects [22,23,24]. 17-βE_2_ interacts with ERs, activating the MAPK and NFκB signaling pathways and increasing antioxidant activities, like GSH-Px and Mn-SOD, thereby exerting antioxidant effects [25]. Additionally, 17-βE_2_ contains strongly reducing phenolic hydroxyl groups that interact with superoxide anions (O_2_^−^) to reduce their accumulation. These groups can also ionize H^+^ to neutralize hydroxyl radicals (OH^−^), among others. Furthermore, 17-βE_2_ enhances mitochondrial antioxidant capacity and reduces free oxygen radicals (ROS) generation, thereby decreasing oxidative stress-induced cellular damage [26,27,28,29]. Thus, estrogen acts as an antioxidant, scavenging free radicals and contributing to physiological processes such as antioxidative stress, anti-inflammatory responses, and inhibition of apoptosis [30].

The present work analyzed alterations of GSH-Px, SOD, CAT activities, MDA level, and hepatocyte viability under varying 17-βE_2_ concentrations for determining its optimal concentration. Later, alterations of gene level, antioxidase activities (SOD, GSH-Px, CAT), MDA level, hepatocyte viability, and apoptosis treated with 17-βE_2_, non-17-βE_2_, and 17-βE_2_ + ER at various time points were compared under ambient (18 °C) and heat stress challenges (24 °C), which provided important basic data for subsequent whole-transcriptome sequencing analyses of rainbow trout.

## 2. Materials and Methods

### 2.1. Primary Hepatocyte Separation and Culture

The hepatocytes of rainbow trout were separated by our laboratory method [31,32]. For primary culture, rainbow trout primary hepatocytes (six 200 ± 5.5 g healthy rainbow trout livers were collected and separated after mixing) were isolated. All experimental fish were fed a basal diet (purchased from Shandong Hanye Biotechnology Co., Ltd., Rizhao, China) three times a day. The specific treatments are as follows: rainbow trout was administered with 80.0 mg/L anesthetic MS-222 (Sigma Aldrich Co., St. Louis, MO, USA), followed by surface disinfection of the fish using 75% ethanol. The liver tissue was collected in a sterile environment, and hepatocytes were isolated. The density of hepatocytes from the isolation was adjusted and subsequently seeded in 25 cm^2^ cell culture flasks (Corning, Basingstoke, UK) for culture. An optimal high-sugar version of Dulbecco’s Modified Eagle’s Medium (DMEM) served as base medium, which contained 15% fetal bovine serum (FBS, Gibco, Beijing, China), 0.4% L-glutamine, 0.5% 4.5 M NaCl, as well as 1% penicillin–streptomycin (400 IU/mL) and amphotericin (25 μg/mL) for cell culture during incubation. After incubation under 18 °C with 5% CO_2_, cells were subjected to an additional 7 days of culture, and the medium was replenished at 2-day intervals. Following the stabilization of the third generation, subsequent experiments were initiated.

### 2.2. Hepatocyte Glycogen Staining Glycogen Periodic Acid Schiff (PAS) Staining

Primary rainbow trout hepatocytes were cultured to 80% confluence prior to periodic acid-Schiff (PAS) staining in line with specific protocols (Solarbio, Beijing, China). The hepatocytes were fixed using PAS fixative for 15 min before washing with 1× PBS (Solarbio, Beijing, China) per well thrice. A 5 g/L solution of periodic acid was then added, and the mixture was allowed to oxidize for 20 min in the dark under ambient temperature. Following oxidation, distilled water was added to wash the cells twice. Later, Schiff reagent was introduced, and the cells were incubated for 20 min in the dark under ambient temperature. Then, the cells were washed with sodium sulfite solution twice, each lasting 2 min. Finally, the cells were restained with Mayer’s hematoxylin staining solution for 2 min. Afterward, a fluorescence microscope (Olympus IX71, Tokyo, Japan) was employed to observe cells at the magnification of 200×.

### 2.3. Treatment with17-βE_2_

**Figure 1:**
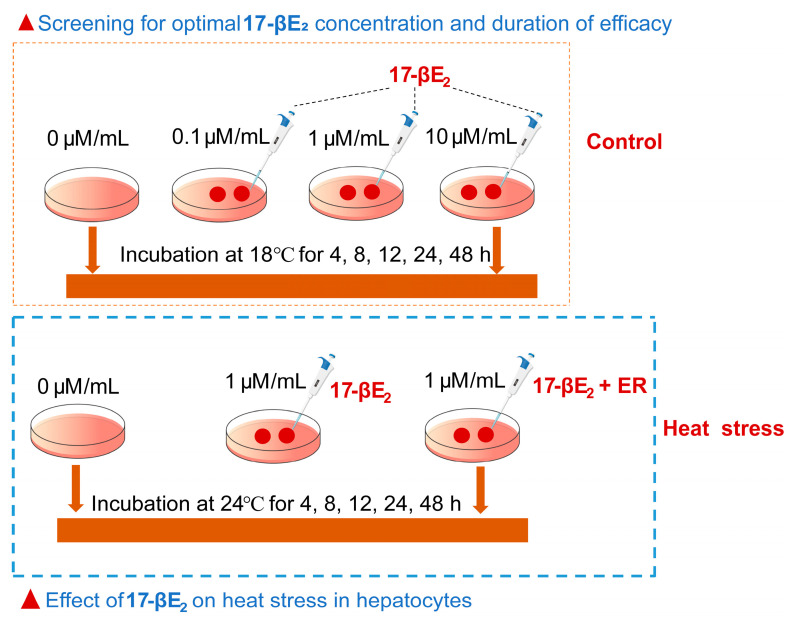
A schematic representation of the experimental procedure.

Hepatocytes (2.5 × 10^5^/well) were cultivated within both 12-well culture plates and 96-well microtiter plates under 18 °C in an incubator with 5% CO_2_. After establishing a dense monolayer of hepatocytes, the cells were divided into four groups and treated with 0, 0.1, 1, or 10 μM/mL of 17-βE_2_ (prepared at 10 mM/mL in DMSO) (Sigma Aldrich Co., St. Louis, MO, USA). Each group underwent incubation for periods of 4, 8, 12, 24, or 48 h, accordingly. Cells from three replicate wells of the same treatment were collected in a 5 mL sterilized centrifuge tube as one sample, and cell samples from 18 wells were collected to obtain six biological replicates. The optimal concentration of 17-βE_2_ for subsequent experiments was identified. A schematic representation of the experimental procedure is shown in Figure 1.

### 2.4. Treatment with 17-βE_2_ and Heat Stress

Hepatocytes were seeded as previously described, cultured in dense monolayers, and classified into 3 groups. The control group included those without 17-βE_2_, the 17-βE_2_ treatment group (determined the optimum concentration), and the 17-βE_2_ + ER ICI182780 (10 mM/mL in DMSO) (Sigma Aldrich Co., St. Louis, MO, USA) group (same concentration as 17-βE_2_). To assess heat stress responses, hepatocytes from the three groups underwent 24 h of incubation under 18 °C (to pre-establish repair and defense mechanisms prior to exposure to elevated temperatures) and were subsequently shifted to 24 °C. During the heat stress stage, we harvested cells (before and following the transition to 24 °C) at intervals of 4, 8, 12, 24, and 48 h following group division to conduct pertinent analyses. Cells from three replicate wells of the same treatment were collected in a 5 mL sterilized centrifuge tube as one sample; a total of six samples were collected per experimental group as six biological replicates.

### 2.5. Cell Viability Assay

To assess the viability of hepatocytes, we employed a Cell Counting Kit-8 (CCK-8) assay (Solarbio, Beijing, China). Specifically, digested hepatocyte suspensions were inoculated into 96-well culture plates at 100 μL/well, achieving a cell density of 2.5 × 10^5^ cells per well. For the 17-βE_2_ concentration screening assay, hepatocytes were treated with varying concentrations of 17-βE_2_ and cultured in a low-temperature incubator (18 °C) for 4, 8, 12, 24, and 48 h. Afterward, CCK-8 reagent (10 μL) was introduced into every well for another 2 h of incubation. Cell viability was subsequently quantified with the microplate spectrophotometer (VARIOSKAN LUX, Thermo Fisfer, Waltham, MA, USA) at 450 nm. For the heat stress experiments, the optimal concentration of 17-βE_2_ identified in the above screening was added to treat hepatocytes with a combination of 17-βE_2_ and ER. After 4, 8, 12, 24, or 48 h of incubation, CCK-8 reagent (10 μL) was introduced into every well, then the mixture was subjected to 2 h of incubation. Finally, cell viability was quantified again using the microplate spectrophotometer (VARIOSKAN LUX, Thermo Fisfer, Waltham, MA, USA) at 450 nm.

### 2.6. Oxidative Stress Indicator Testing

To detect antioxidant enzyme activity, the activities of superoxide dismutase (SOD), glutathione peroxidase (GSH-Px), catalase (CAT), as well as malondialdehyde (MDA) levels were determined with corresponding kits (Nanjing jiancheng, Nanjing, China). In the estrogen concentration screening assay, hepatocytes treated with 0, 0.1, 1, and 10 µM/mL 17-βE_2_ were harvested for 10 min of centrifugation at 1000× *g* and 4 °C. For heat stress experiments, hepatocytes from the non-17-βE_2_, 17-βE_2_, and 17-βE_2_ + ER groups were harvested for 10 min of centrifugation (1000× *g* and 4 °C). The supernatants and hepatocytes from each sample were collected to determine individual oxidase indicators. During testing, hepatocytes were disrupted using ultrasonic fragmentation in an ice-water bath, set to a power of 150 W, with intervals of 3–5 s for a total of 4 cycles lasting 30 s each. Data were acquired using a multifunctional enzyme interpreter (VARIOSKAN LUX, Thermo Fisfer, Waltham, MA, USA).

### 2.7. Apoptosis Detection

The hepatocyte apoptosis rate was assessed with an Annexin V-FITC/PI kit (Beyotime, Shanghai, China). In the heat stress experiments, we incubated cells under 24 °C for a 24 h duration (optimal duration for heat stress during pre-screening). Following various treatments, hepatocytes were collected and washed with PBS before 5 min of centrifugation at 1500× *g*. Precipitated cells were resuspended within a binding buffer containing PI and Annexin V-FITC (5 μL, respectively, a 1:1 ratio) and incubated under 4 °C in the dark for a 30 min duration before analyzing the apoptosis rate by flow cytometry (BD Biosciences, San Diego, CA, USA), utilizing excitation wavelengths of 488 nm and 535 nm, respectively. WinMDI 2.9 software was applied in data analysis.

### 2.8. Ultrastructural Observation

To investigate the effects of heat stress and 17-βE_2_ on cell morphology, this study conducted transmission electron microscopy (TEM). After corresponding treatment, cells first underwent 4 h of fixation with 2.5% glutaraldehyde and were later washed thrice with 0.1 M PBS (pH 7.4) to remove the residual fixative. Next, 1% osmium tetroxide was added to fix cells under ambient temperature for a 2 h period to enhance the contrast of the cell structure. After an ethanol gradient dehydration treatment, cells were polymerized in acetone at 60 °C for 48 h to enhance structural stability. Upon completion of polymerization, cells were sliced into 60 nm thick ultrathin sections using an ultrathin slicer and stained with uranium–lead double staining to highlight internal cellular structures. After staining, section drying was completed under ambient temperature overnight, followed by observation by transmission electron microscopy to capture images in subsequent analyses.

### 2.9. RNA Isolation and qRT-PCR Assay

Total hepatocyte RNA was obtained after various treatments with AG RNAex Pro (AG Biotech Co., Ltd., Changsha, China), following guidelines provided by the manufacturer. A NanoPhotometer spectrophotometer (IMPLEN, Los Angeles, CA, USA) was utilized at a wavelength of 260/280 nm to assess the RNA purity. Total RNA integrity was assessed through 1.0% agarose gel electrophoresis. RNA samples underwent reverse transcription into cDNA with Evo M-MLV RT Kit II (AG Biotech Co., Ltd., Changsha, China). Each sample underwent a quantitative real-time polymerase chain reaction (qRT-PCR) three times using the LightCycler^®^ 480 Instrument II (Roche, Basel, Switzerland) with SYBR^®^ Green Premix Ex Taq HS qPCR Kit (AG Biotech Co., Ltd., Changsha, China), maintaining a 20 µL reaction system. Primer 5.0 software was adopted for developing specific primers (Table S1), with the housekeeping gene β-actin (beta-actin) being a reference for normalization due to its expression stability across the tested temperature range as observed in preliminary experiments. The qRT-PCR protocol involved 30 s under 95 °C, 40 amplification cycles with 5 s denaturation under 95 °C, and 30 s annealing/extension under 60 °C. Amplification specificity was validated based on the melting curve profile. The relative gene expression levels were computed by the 2^−ΔΔCt^ approach.

### 2.10. Statistical Analysis

Data analysis was completed with SPSS software (version 24.0; IBM, Armonk, NY, USA). The Shapiro–Wilk and Levene’s tests were used to examine the normal distribution (*p* > 0.05) and homogeneity of variance (*p* > 0.05), respectively. Inter-group differences were analyzed using one-way analysis of variance (ANOVA). Data were represented by means ± standard deviations (SDs), with each experiment comprising six replicates (n = 6), and the level of significance was equal to 0.05. GraphPad Prism software (version 9.3.1; San Diego, CA, USA) was adopted for graph generation.

## 3. Results

### 3.1. Morphological Observations of Primary Hepatocytes

**Figure 2:**
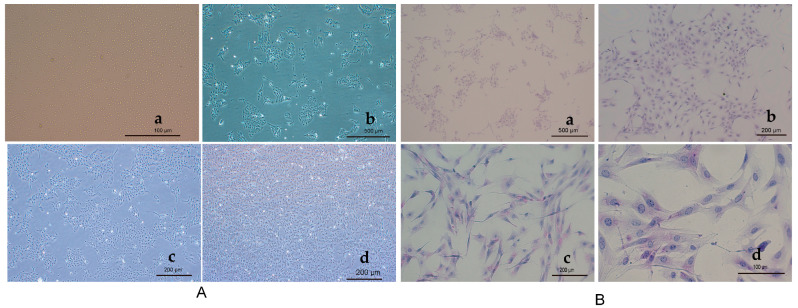
(**A**) Morphological observations on the primary hepatocytes of rainbow trout at different periods of time. Fresh rainbow trout hepatocytes (**a**, 40×), hepatocytes after 2 days of incubation (**b**, 40×), 4-day hepatocytes (**c**, 40×), 6-day hepatocytes (**d**, 40×) of rainbow trout. (**B**) Primary hepatocytes subjected to PAS staining for glycogen. Observation on 40× (**a**), 100× (**b**), 200× (**c**), 400× (**d**).

Rainbow trout hepatocyte morphology at various time points is illustrated in Figure 2. Freshly isolated rainbow trout hepatocytes, digested using 0.25% trypsin, appeared transparent and round (Figure 2(Aa)). Two days later, the majority of hepatocytes had adhered to the plate and transformed from spherical to a flattened shape, with distinct nuclei visible (Figure 2(Ab)). By 4 days, the cells exhibited irregular fibrous shapes and extensive proliferation (Figure 2(Ac)). By 6 days, the cells had spread across the culture flasks and were ready for further culture (Figure 2(Ad)). In the liver, glycogen is stored in granular form in the cytoplasm of hepatocytes. Hepatocytes from rainbow trout were subsequently identified by the PAS staining. Figure 2 shows that glycogen is stained purplish-red and is distributed in a homogeneous or granular pattern (Figure 2(Ba–d)). Thus, PAS staining revealed that the isolated cells were primary hepatocytes.

### 3.2. Effects of 17-βE_2_ on Hepatocyte Viability

To investigate the impact of 17-βE_2_ on hepatocytes derived from rainbow trout, various concentrations of 17-βE_2_ were introduced to the in vitro cultured hepatocytes. The findings indicated that in the 0.1 µM/mL and 10 µM/mL 17-βE_2_ concentration-treated groups, hepatocyte viability was not significantly changed due to prolonged incubation time compared to the control group. At the 1.0 µM optimal dose, cell viability exhibited an initial increase followed by a decline. After a 12 h incubation period, cell viability notably increased (*p* < 0.05) compared with control and all the remaining treatment groups (Table 1). Moreover, the rise in hepatocyte viability over time for the 1.0 µM/mL group was recorded at increments of 14%, 27%, 77%, 48%, and 38% above that of the control group, respectively.

### 3.3. Effects of 17-βE_2_ on the Thermal Stress Parameters

**Figure 3:**
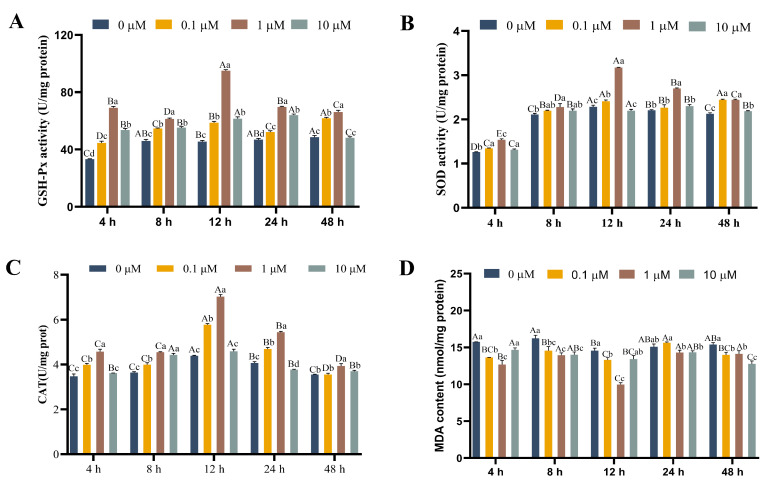
Functions of 17-βE_2_ at varying concentrations in GSH-Px (**A**), SOD (**B**), CAT activities (**C**) (U/mg protein), and MDA level (**D**) (nmol/mg protein) of rainbow trout primary hepatocytes. Data are represented by means ± SD (n = 6). Differences were analyzed using LSD and Duncan’s multiple-range test of one-way ANOVA. The lowercase stands for the difference among concentrations at one time point, while the capital indicates the identical 17-βE_2_ concentration between different time points. (**A**) Normal distribution *p* = 0.258, homogeneity of variance *p* = 0.442; (**B**) normal distribution *p* = 0.208, homogeneity of variance *p* = 0.171; (**C**) normal distribution *p* = 0.239, homogeneity of variance *p* = 0.082; (**D**) normal distribution *p* = 0.098, homogeneity of variance *p* = 0.724.

Changes in SOD, GSH-Px, and CAT activities, as well as MDA biochemical parameters within rainbow trout hepatocytes in response to different concentrations of 17-βE_2_, can be observed in Figure 3. Compared with the control group (0 µM) without heat stress (18 °C), SOD, GSH-Px, and CAT activities increased gradually as estrogen concentration and time elevated and later declined to stable levels, with the highest activity occurring at an effective concentration of 1.0 µM. Notably, SOD, GSH-Px, and CAT levels markedly increased (*p* < 0.05) compared with other groups at 12 h at a concentration of 1.0 µM. With increasing time, the GSH-Px activity increased by 107.9%, 33.4%, 108.4%, 48.8%, and 36.0%, respectively, relative to the control group. In addition, SOD activity increased by 21.8%, 7.9%, 39.0%, 22.4%, and 15.5%, respectively. Moreover, the CAT activity increased by 31.5%, 25.0%, 59.8%, 33.9%, and 10.8%, respectively. However, MDA levels apparently decreased (*p* < 0.05) in every group compared with the control group (0 µM), and 0.1 µM 17-βE_2_ at 24 h elevated relative to the control group, but there was no significance. Also, 1 µM 17-βE_2_ had the lowest level at 12 h, with a decrease of 5.3%.

### 3.4. Functions of 17-βE_2_ in Thermal Stress-Related Gene Expression

**Figure 4:**
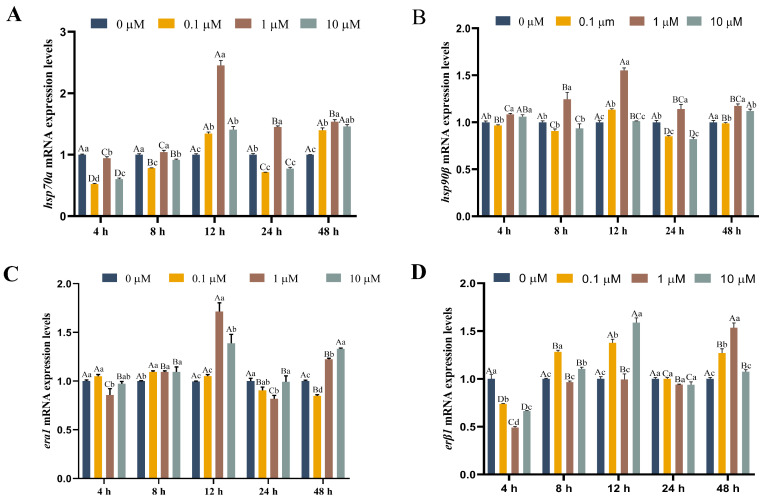
Function of 17-βE_2_ at varying concentrations in *hsp70a* (**A**), *hsp90β* (**B**), *era1* (**C**), and *erβ1* (**D**) expression over time in the absence of heat stress. Data represent means ± SD (n = 6). Differences were analyzed using LSD and Duncan’s multiple-range test of one-way ANOVA. The lowercase stands for the difference among concentrations at one time point, while the capital indicates the identical 17-βE_2_ concentration between different time points. (**A**) Normal distribution *p* = 0.891, homogeneity of variance *p* = 0.109; (**B**) normal distribution *p* = 0.156, homogeneity of variance *p* = 0.954; (**C**) normal distribution *p* = 1.000, homogeneity of variance *p* = 0.580; (**D**) normal distribution *p* = 0.513, homogeneity of variance *p* = 0.075.

Changes in *hsp70a* and *hsp90β* levels, as well as the estrogen receptors *era1* and *erβ1*, are shown in Figure 4 within heat-stressed (18 °C) rainbow trout hepatocytes under 17-βE_2_ treatment. Relative to the control group, *hsp70a*, *hsp90β*, and *era1* expression increased by 145%, 55%, and 71.9% with 1.0 μM 17-βE_2_ at 12 h. However, the expression of *erβ1* was not significantly different between each concentration at 24 h (Figure 4D).

### 3.5. Functions of 17-βE_2_ in Heat-Stressed Hepatocyte Viability

**Figure 5:**
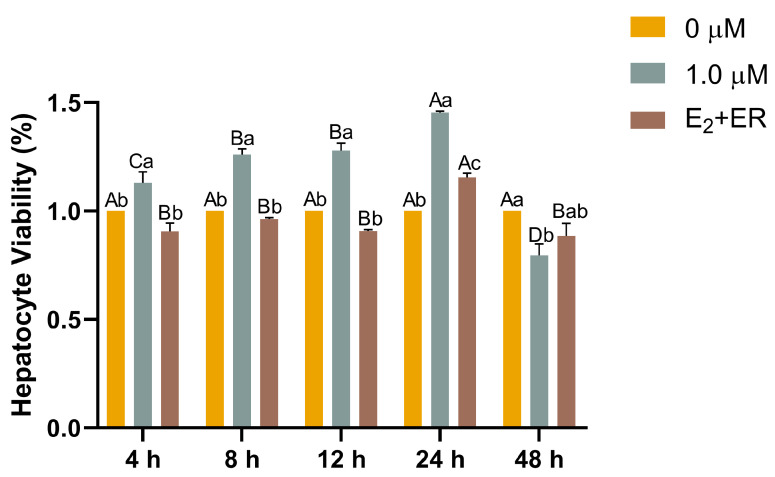
Effects of 17-βE_2_ on primary hepatocyte viability upon heat stress. Data represent means ± SD (n = 6). Differences were analyzed using LSD and Duncan’s multiple-range test of one-way ANOVA. The lowercase stands for the difference among concentrations at one time point, while the capital indicates the identical 17-βE_2_ concentration between different time points. Normal distribution *p* = 0.087, homogeneity of variance *p* = 0.125.

To investigate how 17-βE_2_ affected cell viability upon heat stress, we exposed rainbow trout hepatocytes to screened optimal estrogen concentration of 1.0 μM, 17-βE_2_ + ER (1.0 μM), and subjected the cells to 4, 8, 12, 24, and 48 h of heat stress. Hepatocyte viability tended to increase and then decrease with increasing duration of heat stress in the 1.0 μM 17-βE_2_ relative to the control groups (normalized to control), with increases of 13%, 26%, 28%, and 45% at 4, 8, 12, and 24 h, respectively, peaking at 24 h. However, when estrogen was cotreated with its receptor, cell viability decreased relative to the control group at 24 h, and although it increased by 24 h, it did not reach the levels observed in the group treated with estrogen alone (Figure 5).

### 3.6. Functions of 17-βE_2_ and Heat Stress in the Thermal Stress Parameters

**Figure 6:**
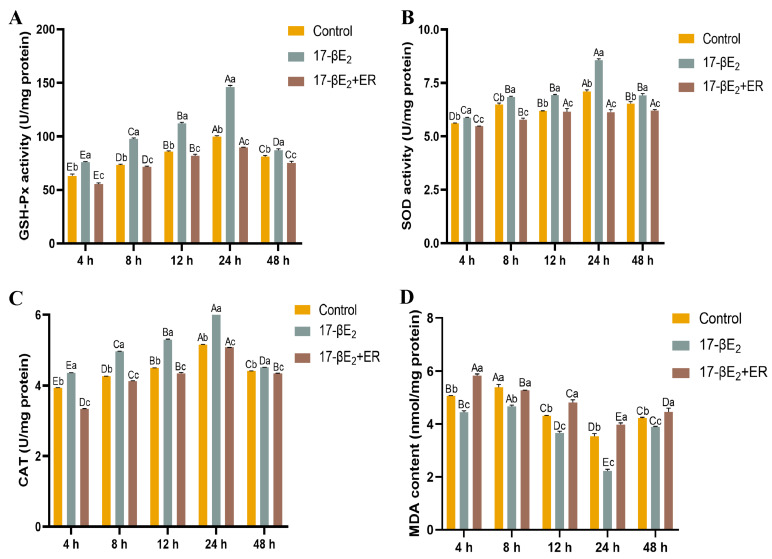
Effects of 17-βE_2_ on GSH-Px (**A**), SOD (**B**), CAT activities (**C**), and MDA level (**D**) in heat-stressed rainbow trout primary hepatocytes with time. Data represent means ± SD (n = 6). Differences were analyzed using LSD and Duncan’s multiple-range test of one-way ANOVA. The lowercase stands for the difference among concentrations at one time point, while the capital indicates the identical 17-βE_2_ concentration between different time points. (**A**) Normal distribution *p* = 0.404, homogeneity of variance *p* = 0.384; (**B**) normal distribution *p* = 0.103, homogeneity of variance *p* = 0.229; (**C**) normal distribution *p* = 0.068, homogeneity of variance *p* = 0.102; (**D**) normal distribution *p* = 0.113, homogeneity of variance *p* = 0.164.

To analyze how 17-βE_2_ affected antioxidant enzymes within rainbow trout hepatocytes under heat stress, we classified cells into three groups, namely, the control group (culture medium), 17-βE_2_ group (1.0 μM optimal concentration), and 17-βE_2_ + ER group, and SOD, GSH-px, CAT activities, and the MDA contents within rainbow trout hepatocytes subjected to different treatments were assayed. As illustrated in Figure 6, GSH-px activity was significantly elevated during heat stress in the 17-βE_2_ group compared to the control group, showing increases of 20.1%, 33.3%, 30.6%, 46.4%, and 7.3% (*p* < 0.05) over time. Conversely, in the 17-βE_2_ + ER group, GSH-px activity decreased by 11.5% at 24 h of heat stress (Figure 6A). SOD activity exhibited increases of 4.45%, 5.55%, 12.14%, 20.70%, and 6.13% (*p* < 0.05) over time, while it decreased by 15.8% in the 17-βE_2_ + ER group at 24 h of heat stress (Figure 6B). CAT activity rose by 10.63%, 16.36%, 17.78%, 19.48%, and 2.32% (*p* < 0.05) but declined by 1.6% in the 17-βE_2_ + ER group at 24 h of heat stress (Figure 6C). In contrast, MDA content decreased progressively by 13.9%, 15.4%, 17.5%, 58.9%, and 8.7%, whereas in the 17-βE_2_ + ER group, MDA content increased with prolonged heat stress duration in comparison with the control group (Figure 6D).

### 3.7. Effects of 17-βE_2_ and Heat Stress on Thermal Stress-Related Gene Expression

**Figure 7:**
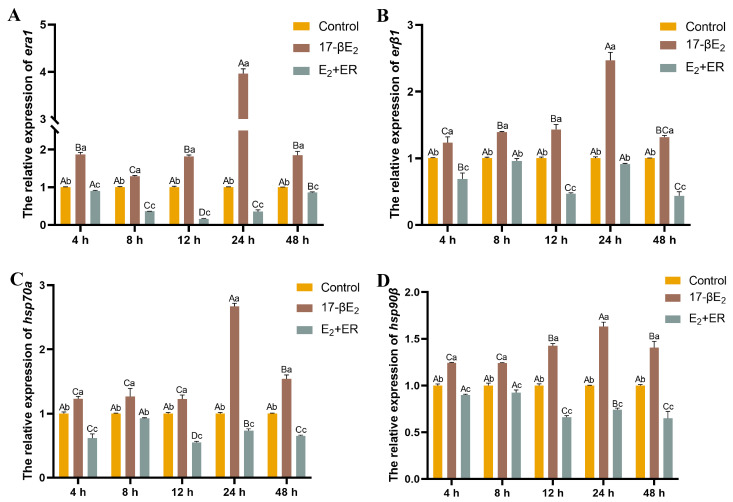
Effects of 17-βE_2_ on gene expression of *era1* (**A**), *erβ1* (**B**), *hsp70a* (**C**), and *hsp90β* (**D**) in heat-stressed rainbow trout primary hepatocytes. Data represent means ± SD (n = 6). Differences were analyzed using LSD and Duncan’s multiple-range test of one-way ANOVA. The lowercase stands for the difference among concentrations at one time point, while the capital indicates the identical 17-βE_2_ or 17-βE_2_ + ER concentration between different time points. (**A**) Normal distribution *p* = 0.927, homogeneity of variance *p* = 0.301; (**B**) normal distribution *p* = 0.589, homogeneity of variance *p* = 0.119; (**C**) normal distribution *p* = 0.580, homogeneity of variance *p* = 0.179; (**D**) normal distribution *p* = 0.840, homogeneity of variance *p* = 0.104.

The optimal 17-βE_2_ concentration (1.0 μM/mL) was added to treat hepatocytes. In order to explore how 17-βE_2_ and heat stress affected heat stress-related gene expression within rainbow trout liver cells, this study detected the expression levels of *era1*, *erβ1*, *hsp70a*, and *hsp90β*. Data were normalized to the control (0 μM/mL, 24 °C). As shown in Figure 7, compared with heat stress, 17-βE_2_ significantly increased associated gene expression.

### 3.8. Effects of 17-βE_2_ on Hepatocyte Histopathological Alterations upon Heat Stress

**Figure 8:**
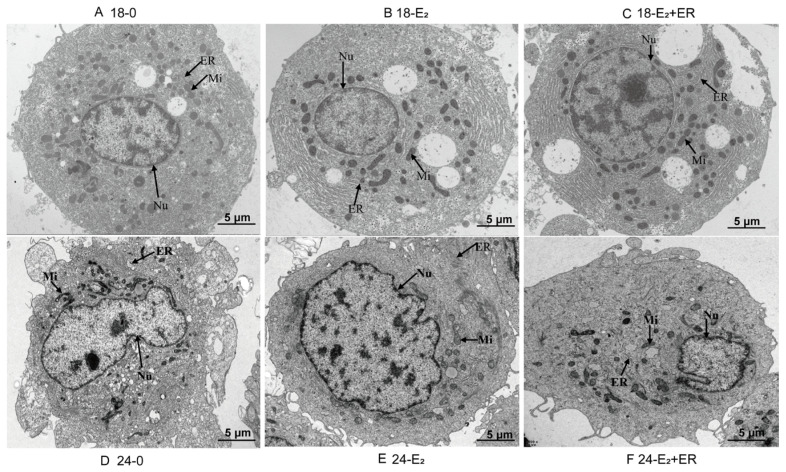
TEM images showing rainbow trout hepatocytes in the control group at 18 °C (**A**), 17-βE_2_ group at 18 °C (**B**), 17-βE_2_ + ER group at 18 °C (**C**), control group at 24 °C (**D**), 17-βE_2_ group at 24 °C (**E**), 17-βE_2_ + ER group at 24 °C (**F**). Nu, nucleus; Mi, mitochondrion; ER, endoplasmic reticulum. Scale bar: 5 μm.

For assessing the protection of 17-βE_2_ for heat-stressed hepatocytes, transmission electron microscopy (TEM) was conducted to observe ultrastructural alterations. Figure 8 demonstrates that cell structures were normal in the 18-0, 18-E_2_, and 18-E_2_ + ER groups, with regular nuclear shapes, intact and evenly distributed mitochondrial membranes, no significant rupture, relatively clear mitochondrial cristae, and no noticeable expansion of the endoplasmic reticulum (Figure 8A–C). Conversely, in the heat stress group, varying degrees of damage were observed; the cell structures appeared abnormal, mitochondrial volumes were reduced, and some mitochondrial membranes were damaged. The endoplasmic reticulum exhibited varying degrees of expansion. The 24-E_2_ + ER group showed the most severe damage, followed by the 24-0 group, while 24-E_2_ caused minimal damage (Figure 8D–F).

### 3.9. 17-βE_2_ Reduces Heat Stress-Mediated Rainbow Trout Hepatocyte Apoptosis

**Figure 9:**
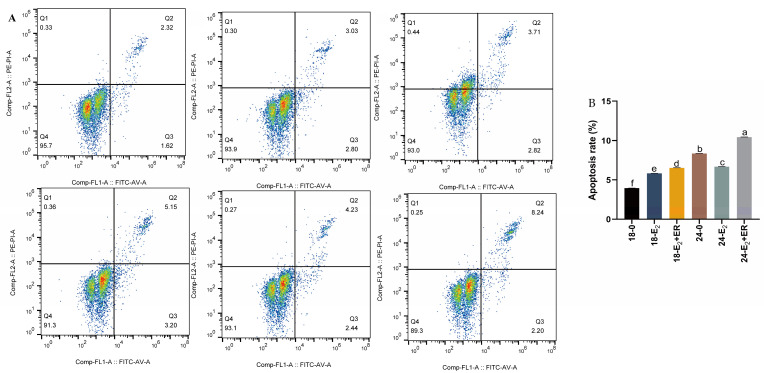
17-βE_2_ effected on heat stress-induced apoptosis in rainbow trout hepatocytes. (**A**) Flow cytometry data. (**B**) Quantitative analysis of cell apoptotic rate. Different letters in each panel represent significant differences (*p* < 0.05). Normal distribution *p* = 0.068, homogeneity of variance *p* = 1.000.

To assess how 17-βE_2_ affected rainbow trout hepatocyte apoptosis upon heat stress, apoptosis was detected via flow cytometry (Figure 9A), and the apoptosis rate was determined (Figure 9B). As a result, the cell apoptosis rate was the lowest in the 18-0 group, and that of the 24-0 group was 43.22%, greater than the 18-0 group (*p* < 0.05). In the heat stress group, 24-E_2_-induced apoptosis was the lowest, decreasing by 25.2% and 56.5% compared with that in the 24-0 and 24-E_2_+ER groups (*p* < 0.05), respectively.

## 4. Discussion

Increasing evidence suggests that estrogens have potent antioxidant effects, mainly through receptor-dependent and nonreceptor-dependent pathways [33]. The nonreceptor-dependent pathway involves the presence of a strongly reducing phenolic hydroxyl group in 17-βE_2_, which can interact with the superoxide anion (O_2_^−^) to prevent the accumulation of damaging O_2_^−^ during stress, and the phenolic hydroxyl group can ionize H^+^ and neutralize the hydroxyl radical (OH^−^). Thus, estrogen acts as an antioxidant to scavenge free radicals and is involved in physiological processes such as antioxidative stress, anti-inflammatory, and inhibition of apoptosis [30]. Receptor-dependent pathways operate through the nuclear estrogen receptor (ER). Typically, estrogens induce transcriptional genomic effects via ERs within the nucleus. Estrogen-bound ERs are ligand-dependent transcription factors that can interact with estrogen-responsive elements to activate specific genes within estrogen-responsive tissues [34]. To explore why female rainbow trout exhibit greater heat tolerance than males, primary cultured rainbow trout hepatocytes were treated with various concentrations of 17-βE_2_ and exposed to heat stress. The expression trends of heat shock protein genes, estrogen receptor genes, and antioxidant enzymes were then monitored.

High temperature causes heat stress in rainbow trout, probably associated with oxidative stress [35,36]. After the body is stimulated by high temperature, excessive ROS production results in metabolite generation and accumulation, like MDA, which interferes with the normal redox balance and ultimately destroys biological macromolecule activity and structure, including nucleic acids, proteins, and lipids, causing oxidative stress in the body [37,38,39]. To reduce oxidative damage, the body employs its antioxidant system to eliminate excess ROS. This system mostly consists of SOD, GPx, CAT, as well as additional related enzymes [40]. GPx facilitates glutathione (GSH) reduction to oxidized glutathione (GSSG), aiding in H_2_O_2_ breakdown, which helps in scavenging H_2_O_2_ and reducing lipid peroxidation. SOD transforms superoxide into O_2_ and H_2_O_2_, and CAT can later decompose H_2_O_2_ to O_2_ and H_2_O_2_, thus mitigating oxygen radical damage and decreasing lipid peroxidation [41]. According to our results, estradiol addition improved hepatocyte viability in primary rainbow trout (Table 1). Additionally, cell viability reached its peak at 24 h under heat stress (Figure 5), which apparently was elevated in comparison with the control group (*p* < 0.05). Relative to the control group, increasing the 17-βE_2_ treatment dose and duration led to a rise and subsequent decline in SOD, GSH-Px, and CAT activities, while MDA content initially fell and then increased (Figure 3). This study demonstrated that 17-βE_2_ has a positive effect on antioxidant enzyme activity, which reduces MDA content. The GSH-Px activity at the 1.0 μM/mL 17-βE_2_ dose was significantly greater (*p* < 0.05) than that at the other doses and for 12 h. The SOD activity peaked in the 1.0 μM/mL 17-βE_2_ group at 12 h. Moreover, compared with the other groups, the 1.0 μM/mL 17-βE_2_ group presented highly significant (*p* < 0.05) differences at 12 h (Figure 3). GSH-Px, CAT, and SOD activities were reduced by 10 μM/mL 17-βE_2_, probably associated with a reduction in cellular activity and estrogen-induced cytotoxicity at high estrogen concentrations. This study suggested that estradiol can increase SOD, GSH-Px, and CAT activities and decrease the MDA level within rainbow trout hepatocytes dose-dependently, which improves the antioxidation capacity of rainbow trout heat stress, reduces oxidative damage in vivo, and protects hepatocytes against metabolic diseases due to heat stress. Estrogen interacts with the ER to activate mitogen-activated protein kinase signaling pathways such as the MAPK and nuclear factor kappa B (NFκB) pathways and increases antioxidant enzyme activity such as GSH-Px and manganese superoxide dismutase (Mn-SOD), thereby exerting antioxidant effects on estrogen.

Additionally, HSPs are highly conserved biologically, exhibit molecular chaperone activity, and play roles in protein biosynthesis as well as transmembrane translocation. They function to prevent intracellular protein denaturation, minimize protein misfolding, enhance misfolded protein repair, maintain cellular homeostasis, and ensure structural integrity [32,42]. We found that *hsp70a* and *hsp90β* expression of the 17-βE_2_ group was remarkably elevated relative to the control group. Notably, at the same 17-βE_2_ concentration (1.0 μM/mL), *hsp70a* and *hsp90β* levels of the heat stress group (24 °C) were elevated compared with the non-heat stress group (18 °C) at 24 h. Furthermore, *era1* expression increased in the heat-stressed group (24 °C) compared to the non-heat-stressed group (18 °C) at 24 h. Numerous studies have shown that HSP90 and HSP70 can bind to the ER, the receptor for estrogen 17-βE_2_, and assemble into chaperonin complexes that stabilize HSPs in an inactive state. Several studies have shown that under estrogen-deficient conditions, HSPs tend to form complexes with estrogen receptors, resulting in the impaired molecular chaperone activity of HSPs and the inhibition of their heat shock protective function. In contrast, in an estrogen-sufficient environment, the estrogen receptor dissociates from the HSP-ER complex, thereby releasing HSPs and enabling them to regain their heat shock protective effects. In addition, this dissociation triggers an estrogen-mediated signaling cascade that enhances the adaptive cellular response to heat shock [43,44]. This can also explain our previous findings that the “estrogen signaling pathway is an important pathway enriched by differential genes and differential proteins before and following heat stress”. The physiological response mechanism in rainbow trout to thermal stimuli involves the up-regulation of heat stress proteins (HSPs) [45]. This response not only enhanced the trout’s capacity to adapt to thermal changes but also activated the antioxidant enzyme system in the body [46]. Through this mechanism, an effort is made by rainbow trout to preserve the dynamic balance between oxidative and antioxidative systems to counteract potential damage from heat stress. Additionally, pretreatment with 17-βE_2_ in a trauma/hemorrhagic shock rat model prevented reductions in the HSP60 and HSP90 levels in the heart and increased HSP32 expression, a process mediated by AKT activation [47].

Rainbow trout (*Oncorhynchus mykiss*) is a cold-water species that plays a significant role in scientific research and practical production as a model organism for heat stress studies. However, conducting experiments with live rainbow trout presents several challenges. Firstly, due to their high oxygen demand and stringent water quality requirements, maintaining a constant flow condition, which is essential for precise temperature control in heat stress research, is difficult to achieve. Secondly, raising fish under laboratory conditions that meet precise temperature control requirements may introduce various stressors beyond heat stress, such as hunger stress and fishing stress. Cell culture in vitro technology has become an indispensable tool in modern life sciences, particularly in the study of gene function and physiological mechanisms. Primary fish hepatocytes are commonly utilized as an in vitro model to investigate how exogenous stress induces injury [48]. The liver exerts an important effect on estrogen synthesis in fish; however, while hepatocytes cannot directly synthesize estrogen, the liver produces vitellogenin and zona pellucida proteins, which are crucial precursor proteins for estrogen synthesis [49]. Vitellogenin expression in rainbow trout is regulated by the endoplasmic reticulum via the classical endoplasmic reticulum pathway [50]. Cells exposed to heat stress exhibit notable changes in internal structure, including alterations in nuclear morphology, a reduction in mitochondrial number, and damage to mitochondrial structure, which are key indicators of the cellular stress response [51]. In the present study, heat stress led to wrinkling and shrinkage of the nuclei in rainbow trout hepatocytes, a reduction in mitochondrial number, increased bilayer membrane density, and vacuolization or rupture of mitochondrial crista structures. Nonetheless, supplementation with 17-βE_2_ alleviated these changes, indicating a protective effect from heat stress-mediated hepatocellular damage. The function of estrogen in antagonizing heat stress is previously explored in zebrafish, Mozambique tilapia [52], and mummichog (*Fundulus heteroclitus*) [53], yielding similar results, thereby supporting the validity of the current research model for future applications.

Heat stress causes cellular mitochondria to produce peroxides that damage the organism, leading to apoptosis [54]. Apoptosis accounts for the orderly, autonomous, and genetically controlled cell death, which can maintain homeostasis [55]. Flow cytometry and CCK-8 further demonstrated that, compared with control fish, heat stress-treated fish presented a greater rate of apoptosis. Based on these results, heat stress causes rainbow trout hepatocyte apoptosis. However, the apoptosis rate of hepatocytes treated with 1.0 μM/mL 17-βE_2_ and then exposed to heat stress remarkably decreased relative to the heat-stressed group without estrogen treatment, which exhibited up-regulated expression of the heat shock proteins HSP70 and HSP90. One study revealed that both HSP70 and HSP90 inhibited JNK-mediated cell death through direct inhibition of the phosphorylation or inhibition of upstream kinases and decreased Bcl-XL expression (a member of the antiapoptotic Bcl-2 family) [56]. Another study revealed higher levels of HSP72 in cardiac and renal tissues in intact females than in males, both at baseline and after ischemia/reperfusion [57,58]. Additionally, 17-βE_2_ induces the phosphorylation of HSP27 and αβ-crystallin through p38 MAP kinase [59], which is crucial for the protective functions of these proteins. A challenge in the study of estrogen and heat shock protein expression is the variability seen across animal models. Further research is required to understand why heat stress elicits a more pronounced HSP response within female rainbow trout compared to males.

## 5. Conclusions

In summary, HSP expression is enhanced synergistically by heat stress and estrogen. Furthermore, estrogen plays a protective role for cells stressed by high temperatures by stimulating the HSP pathway. These findings elucidate possible protective benefits of estrogen in preventing hepatocyte damage caused by excessive heat stress, indicating that estrogen could serve as a valuable adjunct in combating heat stress.

## Figures and Tables

**Table 1:** Effects of various 17-βE_2_ concentrations and treatment times on hepatocyte viability without heat stress.

Group	0 µM/mL	0.1 µM/mL	1 µM/mL	10 µM/mL
4 h	1.00 ^aA^ ±00	1.12 ^aA^ ±0.11	1.14 ^aE^ ±0.01	1.02 ^aAB^ ±0.15
8 h	1.00 ^bA^ ±00	0.93 ^cB^ ±0.01	1.27 ^aD^ ±0.01	0.85 ^dB^ ±0.00
12 h	1.00 ^dA^ ±00	1.07 ^cAB^ ±0.01	1.77 ^aA^ ±0.02	1.12 ^bA^ ±0.01
24 h	1.00 ^cA^ ±00	1.18 ^bA^ ±0.00	1.48 ^aB^ ±0.01	1.19 ^bA^ ±0.01
48 h	1.00 ^dA^ ±00	1.05 ^cAB^ ±0.01	1.38 ^aC^ ±0.01	1.09 ^bA^ ±0.00

According to the LSD and Duncan’s multiple range test, means ± SD (n = 6) with different lowercase and capital letters in the same row and column were significantly different (*p* < 0.05). The lowercase indicates differences between concentrations at the same time and the capital between times of the same 17-βE_2_ concentration. Normal distribution *p* = 0.373, homogeneity of variance *p* = 0.060.

## Data Availability

The data of this study will be made available upon reasonable request.

## Supplementary Materials

The following supporting information can be downloaded at: https://www.mdpi.com/article/10.3390/antiox13111316/s1, Table S1 Primer informations for qRT–PCR amplification.
