# Correction: Zhao et al. Protective Effects of 17-βE_2_ on the Primary Hepatocytes of Rainbow Trout (*Oncorhynchus mykiss*) Under Acute Heat Stress. *Antioxidants* 2024, *13*, 1316

**DOI:** 10.3390/antiox14060659

**Published:** 2025-05-30

**Authors:** Guiyan Zhao, Zhe Liu, Junhao Lu, Jinqiang Quan, Yucai Pan

**Affiliations:** Department of College of Animal Science and Technology, Gansu Agricultural University, Lanzhou 730070, China; 18893812949@163.com (G.Z.); lujh@st.gsau.edu.cn (J.L.); quanjq@gsau.edu.cn (J.Q.); panyc@st.gsau.edu.cn (Y.P.)

In the original publication [1], there was a mistake in Figure 8E as published. The authors regret that an error occurred in the original version of Figure 8E due to inadvertent misplacement during the figure preparation process. The corrected Figure 8E appears below. The authors state that the scientific conclusions are unaffected. This correction was approved by the Academic Editor. The original publication has also been updated.

## Figures and Tables

**Figure 8:**
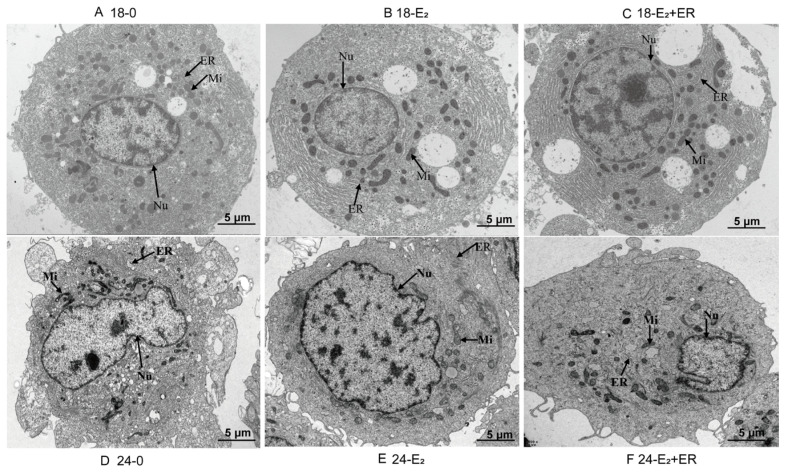
TEM images showing rainbow trout hepatocytes in the control group at 18 °C (**A**), 17-βE_2_ group at 18 °C (**B**), 17-βE_2_ + ER group at 18 °C (**C**), control group at 24 °C (**D**), 17-βE_2_ group at 24 °C (**E**), 17-βE_2_ + ER group at 24 °C (**F**). Nu, nucleus; Mi, mitochondrion; ER, endoplasmic reticulum. Scale bar: 5 μm.
